# An infant mouse model of influenza-driven nontypeable *Haemophilus influenzae* colonization and acute otitis media suitable for preclinical testing of novel therapies

**DOI:** 10.1128/iai.00453-23

**Published:** 2024-04-11

**Authors:** Katherine R. Landwehr, Caitlyn M. Granland, Kelly M. Martinovich, Naomi M. Scott, Elke J. Seppanen, Luke Berry, Deborah Strickland, Alma Fulurija, Peter C. Richmond, Lea-Ann S. Kirkham

**Affiliations:** 1 Wesfarmers Centre of Vaccines and Infectious Diseases, Telethon Kids Institute, Perth, Australia; 2 School of Population Health, Curtin University, Perth, Australia; 3 Wal-yan Respiratory Research Centre, Telethon Kids Institute, Perth, Australia; 4 Centre for Child Health Research, University of Western Australia, Perth, Australia; 5 Department of Paediatrics, School of Medicine, University of Western Australia, Perth, Australia; 6 Department of Immunology, Perth Children’s Hospital, Child and Adolescent Health Service, Perth, Australia; University of Pennsylvania, Philadelphia, Pennsylvania, USA

**Keywords:** otitis media, nontypeable *Haemophilus influenzae* (NTHi), infant mouse model, infection, influenza, preclinical model

## Abstract

Nontypeable *Haemophilus influenzae* (NTHi) is a major otitis media (OM) pathogen, with colonization a prerequisite for disease development. Most acute OM is in children <5 years old, with recurrent and chronic OM impacting hearing and learning. Therapies to prevent NTHi colonization and/or disease are needed, especially for young children. Respiratory viruses are implicated in driving the development of bacterial OM in children. We have developed an infant mouse model of influenza-driven NTHi OM, as a preclinical tool for the evaluation of safety and efficacy of clinical therapies to prevent NTHi colonization and the development of OM. In this model, 100% of infant BALB/cARC mice were colonized with NTHi, and all developed NTHi OM. Influenza A virus (IAV) facilitated the establishment of dense (1 × 10^5^ CFU/mL) and long-lasting (6 days) NTHi colonization. IAV was essential for the development of NTHi OM, with 100% of mice in the IAV/NTHi group developing NTHi OM compared with 8% of mice in the NTHi only group. Histological analysis and cytokine measurements revealed that the inflammation observed in the middle ear of the infant mice with OM reflected inflammation observed in children with OM. We have developed the first infant mouse model of NTHi colonization and OM. This ascension model uses influenza-driven establishment of OM and reflects the clinical pathology of bacterial OM developing after a respiratory virus infection. This model provides a valuable tool for testing therapies to prevent or treat NTHi colonization and disease in young children.

## INTRODUCTION

Nontypeable *Haemophilus influenzae* (NTHi) is a Gram-negative bacterium that exhibits opportunistic pathogenic behavior in humans (1). Asymptomatic nasopharyngeal colonization with NTHi is common and transient in children, with carriage frequencies ranging from ~15% to 100% depending upon age and geographic region (2–5). NTHi can also cause a range of diseases including sinusitis, conjunctivitis, pharyngitis, meningitis, bacteraemia, pneumonia, and otitis media (OM) (6, 7). Globally, NTHi is the predominant pathogen associated with chronic OM with effusion, recurrent acute OM, or acute OM with failure to treat (8). Early and dense NTHi colonization is associated with the development of acute and recurrent OM (6, 9–13). Triggers for the development of asymptomatic NTHi colonization into OM are thought to involve virus-induced inflammation, which permits proliferation and dissemination of NTHi from the nasopharynx along the Eustachian tube and into the middle ear (14, 15).

The burden of OM is large, with over 700 million annual acute OM cases diagnosed globally, 30 million chronic cases, and over 21,000 OM-associated deaths annually. An estimated 51% of all OM is in children under 5 years of age (16). With such a high prevalence in the pediatric population, OM is the main reason that antibiotics are prescribed for pre-school children (17). Unfortunately, antibiotic treatment for OM is often ineffective (18).

Despite major efforts (19–22), there are no licensed vaccines or other therapies that have been proven to prevent NTHi colonization and/or infection (23–25). As development of preventative therapies for NTHi diseases including OM continues, relevant mouse models are important. However, intranasal inoculation of rodents with NTHi does not reliably result in development of NTHi OM (26). Therefore, NTHi OM animal models often involve direct inoculation of NTHi into the middle ear (either via the bulla or through the tympanic membrane) [reviewed in references (27, 28)], which does not accurately reflect the progression of disease in humans. NTHi OM ascension models, where intranasal delivery of a respiratory virus facilitates NTHi ascension from the nose to the ear have been established in chinchillas using adenovirus (29), and in adult mice using influenza (14, 26). These biologically relevant models of disease are most suitable for testing therapies to prevent or treat NTHi OM; especially as preventing dissemination of NTHi from the nose to the ear (or lung, meninges, and blood) is likely to be the most effective strategy for prevention of NTHi disease. As murine models are more accessible than chinchillas (cost, handling, availability, sourcing of animals, and animal-specific reagents), and NTHi OM is mostly a childhood infection, we adapted the adult mouse model of influenza-driven NTHi OM (14) into an infant mouse model. To our knowledge, this is the first infant animal model of NTHi colonization and OM. The model reflects real-world conditions and is suitable for preclinical testing of the safety and efficacy of therapies for prevention or treatment of acute NTHi OM in young children.

Male and female 7-day-old mice were intranasally administered either saline or influenza A virus (IAV). After 3 days, mice were intranasally administered either saline or NTHi. Nasal washes and middle ear and lung tissue samples were collected 5, 7, and 9 days post-treatment and analyzed for bacterial counts, viral titer, and inflammation.

## RESULTS

### Infant mice receiving both influenza and NTHi had dense and persistent NTHi colonization and were more likely to develop NTHi OM compared to mice that did not receive influenza

**Fig 1:**
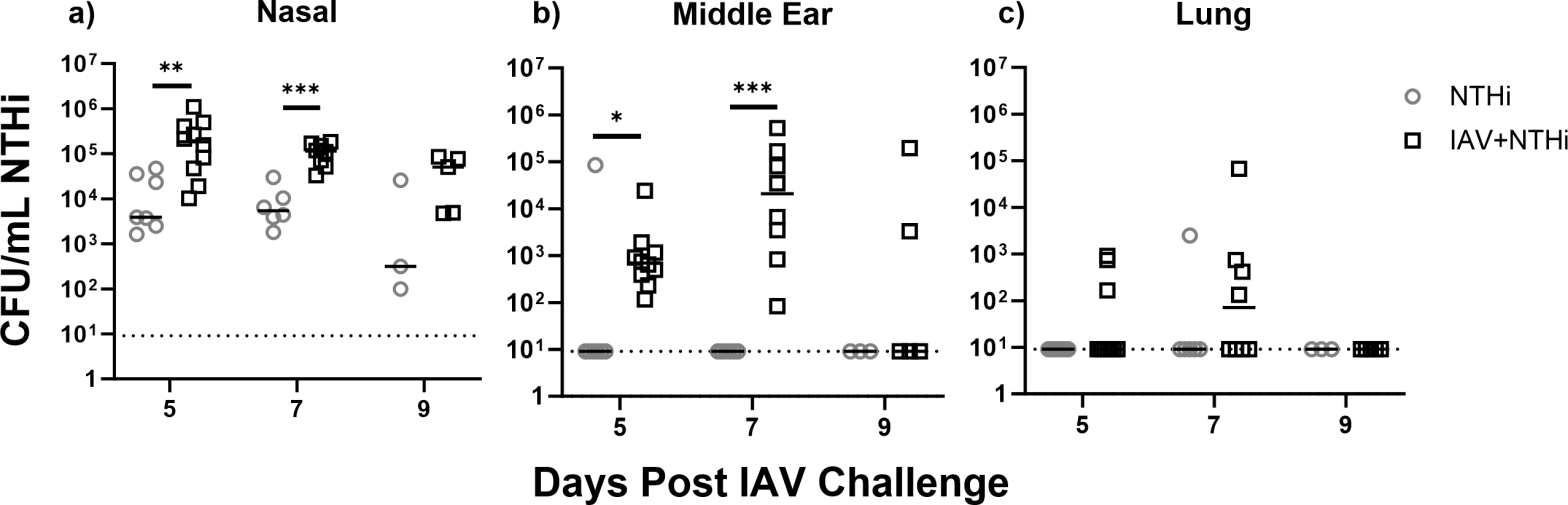
NTHi counts (CFU/mL) in (a) nasal wash, (b) middle ear tissue, and (c) lung tissue on Days 5, 7, and 9, post-IAV challenge. Nontypeable *Haemophilus influenzae* (NTHi) density in log_10_ CFU/mL from (a) nasal wash, (b) middle ear tissue homogenate, and (c) lung tissue homogenate from mice on Days 5, 7, and 9, pos-influenza A (IAV) or saline treatment on Day 0, and NTHi challenge on Day 3. Each dot represents an individual mouse. Horizontal bars depict the median NTHi density, and the dotted line represents the limit of quantification by culture. Open circles represent NTHi titers in the NTHi only treatment group, open squares represent NTHi titers in the IAV + NTHi treatment group. *P < 0.05; **P < 0.01; ***P < 0.001 when compared between treatment groups at the same time point using the Mann-Whitney test.

NTHi colonization was observed in the nasal wash of all mice in the IAV + NTHi and NTHi only groups on Days 5, 7, and 9 (Fig. 1a). NTHi was not detected in the nasal washes from the saline-treated control group or the IAV only group. Median NTHi titers in the nasal washes were 2 LOG higher in the IAV + NTHi challenged mice compared to the NTHi only group (*P* < 0.01 on Day 5, and *P* < 0.001 on Day 7; Fig. 1a). NTHi titers in the nasal wash had a median of 5.08 × 10^4^ CFU/mL on Day 9 in the IAV + NTHi group (a titer that was never reached in the NTHi only group). In contrast, NTHi colonization was clearing by Day 9 in the NTHi only group.

In the middle ear tissue, 18/18 (100%) mice in the IAV + NTHi challenge group developed OM on Day 5 or Day 7 compared with 1/13 (7.69%) in the NTHi only group (Fig. 1b). Median NTHi titers in the middle ear tissue homogenate were below the limit of detection in the NTHi only group on all days. For the IAV + NTHi groups, the middle ears contained 6.95 × 10^2^ CFU/mL NTHi (IQR: 3.5 × 10^2^ to 1.35 × 10^3^ CFU/mL) on Day 5 and peaked on Day 7 with 2.08 × 10^4^ CFU/mL NTHi (IQR: 1.50 × 10^3^ to 1.46 × 10^5^ CFU/mL) (*P* < 0.05 and *P* < 0.001, respectively). By Day 9, OM was resolving in the IAV + NTHi challenge group with 2/5 mice with NTHi detected in the middle ear tissue. The highest median NTHi titer in middle ear tissue homogenate was on Day 7 of the model (4 days post-NTHi challenge IAV + NTHi group), at 2.08 × 10^4^ CFU/mL (IQR: 1.50 × 10^3^ to 1.46 × 10^5^ CFU/mL).

Low titers of NTHi were detected in the lungs from 7/18 mice in the IAV + NTHi group and 1/13 mice in the NTHi only group on Days 5 and 7 (Fig. 1c). Only the IAV + NTHi group on Day 7 had a median titer above the limit of detection—7.12 × 10^1^ CFU/mL (IQR: 9.12 to 6.75 × 10^4^ CFU/mL). NTHi was not detected in the samples collected from saline-treated control or IAV only challenged mice (median titer below limit of detection). No difference was observed between male and female infant mice for NTHi titers detected in any specimen (Fig. S1).

### No weight loss was observed in the infant NTHi OM mouse model

**Fig 2:**
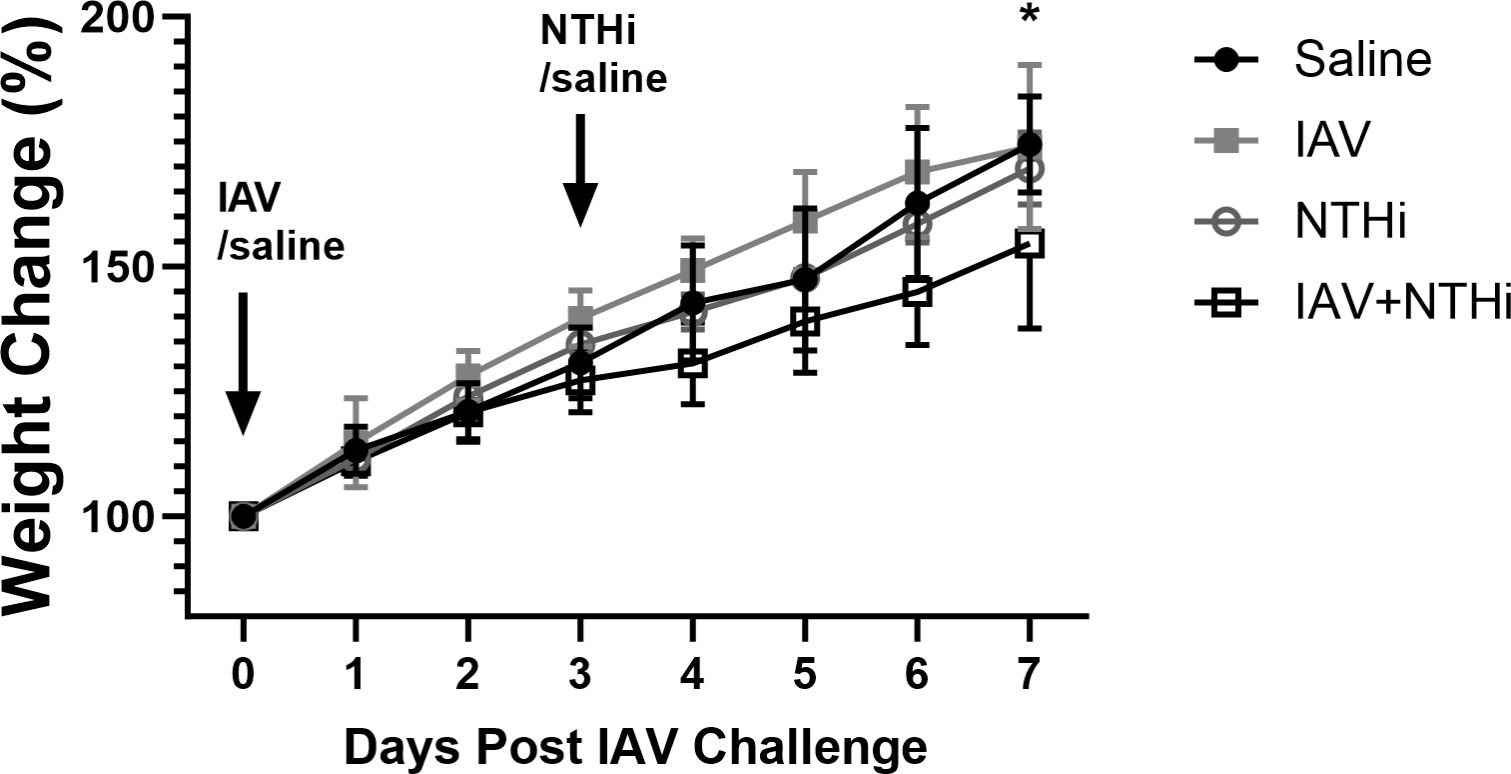
Weight of mice in NTHi OM model percent weight change (mean ± SD) of infant mice intranasally challenged with saline, IAV, NTHi, or IAV + NTHi. Numbers of mice per group decreased over time as samples were collected (Days 1–5, *n* ≥ 6/group; Days 6 and 7, *n* ≥ 3/group). *P < 0.05 when compared between saline and IAV + NTHi-treatment groups on Day 7.

Any weight loss in infant mice is a significant adverse event, from which they are unlikely to recover. No weight loss was observed in this model using an NTHi challenge dose of 5 × 10^5^ CFU (Fig. 2). The IAV + NTHi group had the least weight gain in comparison with the saline control group and the IAV only-treated group (Fig. 2). By Day 7, there was a difference in mean weight gain between the saline only-treated group and the IAV + NTHi group (74.42 ± 9.68% vs 54.63 ± 17.06%, respectively, *P* < 0.05).

While weight loss, or any other clinical symptom, was not observed with a 5 × 10^5^ CFU NTHi challenge dose, a higher NTHi dose of 5 × 10^6^ CFU did result in up to 16% weight loss (Fig. S2a). This was considered too severe an illness for an infant OM model, risking mismothering and mice not surviving until the model ended. In addition, the log-fold increase in NTHi challenge dose did not result in increased NTHi tires in the nasal washes or middle ear of mice on Day 5 or Day 7 of the model (Fig. S2b and c). Thus, an NTHi challenge dose of 5 × 10^5^ CFU was selected for this model [which is 2 LOG lower than the adult mouse NTHi OM model challenge dose of 5 × 10^7^ CFU (14)].

### Influenza A virus was detected in nasal washes, lung tissue, and in some middle ear specimens

**Fig 3:**
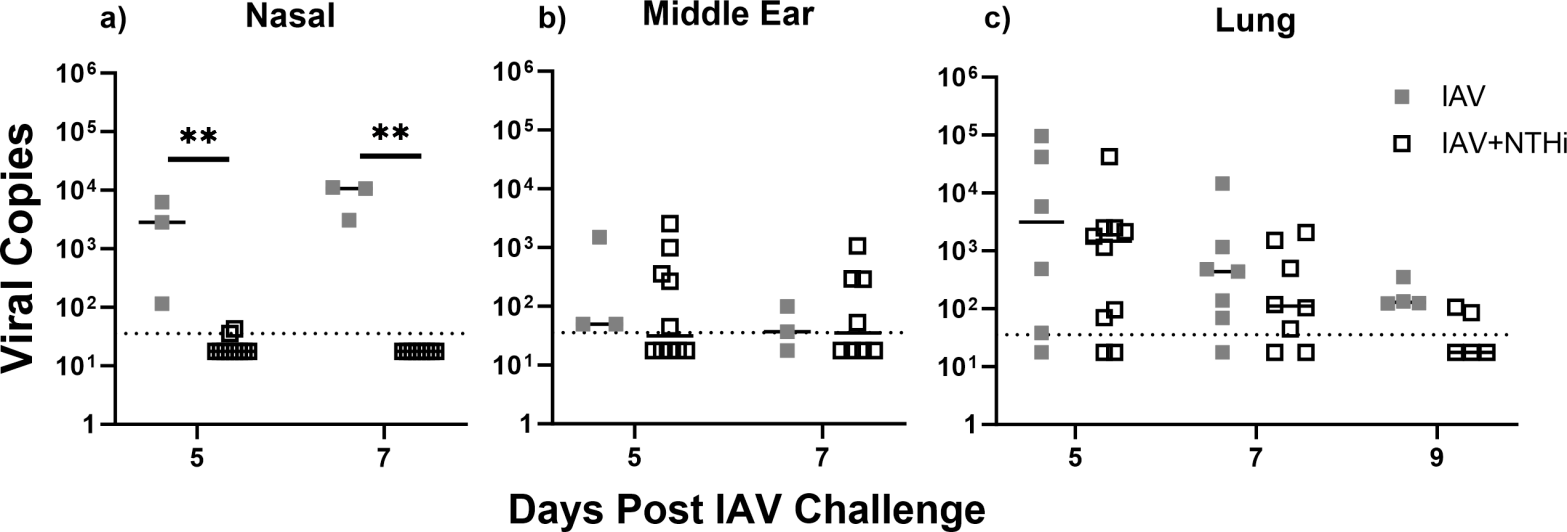
Influenza A virus titers in the nasal wash, middle ear tissue, and lung tissue post-IAV challenge. Influenza A Virus (IAV) titers from (a) nasal wash, (b) middle ear tissue homogenate, and (c) lung tissue homogenate of mice on Day 5, Day 7 (and Day 9, lungs only) post-IAV or IAV + NTHi challenge. Each square represents an individual mouse. Horizontal bars depict the median IAV titer, and the dotted line represents the limit of quantification. **P < 0.01 when compared between treatment groups at the same time point using the Mann-Whitney test.

IAV was detected by qRT-PCR in all of the nasal washes tested in the IAV only-treated mice (*n* = 6 tested; *n* = 3 on Day 5 and *n* = 3 on Day 7) but not the IAV + NTHi group (*n* = 18; *n* = 10 on Day 5 and *n* = 8 on Day 7), with only 2/18 mice in the IAV + NTHi group with a positive IAV PCR, albeit at the limit of detection (Fig. 3a). The median IAV titer in the nasal washes of the IAV group was 2.83 × 10^3^ (IQR: 1.15 × 10^2^ to 6.29 × 10^3^) on Day 5 and 1.06 × 10^4^ (IQR: 1.11 × 10^4^ to 3.06 × 10^3^) on Day 7, higher in comparison with median IAV titers below the limit of detection for the IAV + NTHi group on both days, *P* < 0.01 (Fig. 3a). In the middle ear tissue, IAV was detected some mice in each of the IAV only and IAV + NTHi groups, with median IAV titers just above or at the lowest limit of detection (Fig. 3b). IAV was detected in the lung tissue of most mice, with the IAV only group having higher median IAV titers than the IAV + NTHi group on Days 5, 7, and 9 (Fig. 3c). On Day 9, IAV tires were only measured in the lung tissue and not the nasal wash and middle ear. Of note is the pattern of reduced IAV titers in the presence of NTHi for the nasal washes (Fig. 3a).

### Immune mediator responses in infant model

**Fig 4:**
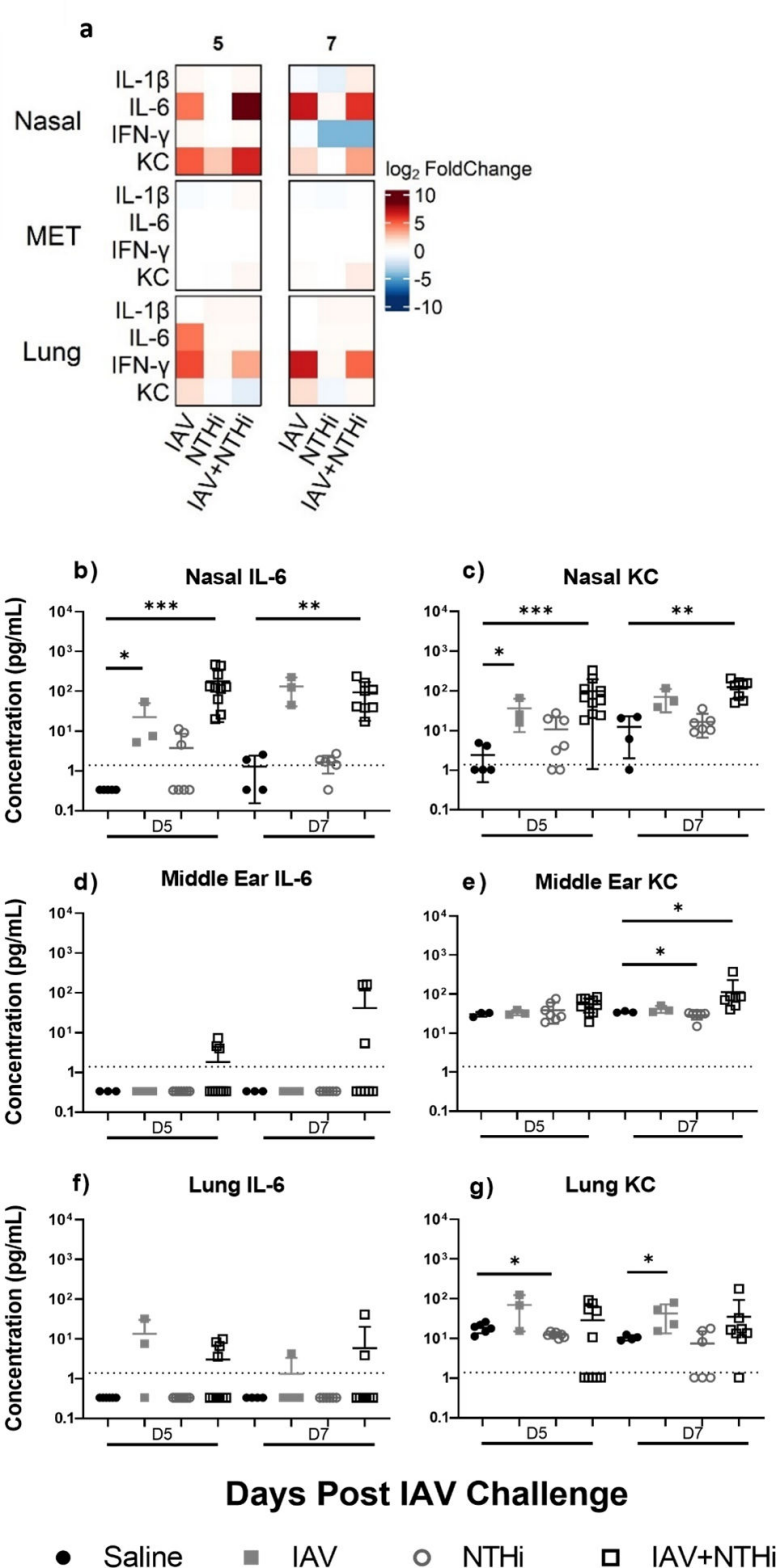
Immune mediator responses in infant NTHi OM model including IL-6 and KC titers in nasal wash, middle ear tissue, and lung on Days 5 and 7 post-IAV challenge. (**a**) Heat map representation of inflammatory mediator levels in nasal washes, and supernatant from middle ear tissue (MET) homogenate and lung tissue homogenate from mice on Days 5 and 7 post-influenza A virus (IAV) or saline treatment. Represented as log_2_ fold change over the saline control mediator response for each analyte for each treatment group. IL-6 and KC titers in nasal washes (**b and c**), middle ear tissue (**d and e**), and lung tissue (**f and g**) from mice on Days 5 and 7 post-IAV challenge or saline treatment. Each dot represents an individual mouse, horizontal bars depict median analyte titer, and the dotted line represents the limit of quantification for each analyte. *P < 0.05; **P < 0.01; ***P  <  0.001 when compared between treatment groups at the same time point using the Mann-Whitney test.

Immune mediators IL-1β, IL-6, IL-10, IFN-γ, and KC were measured in the nasal wash and supernatant from the middle ear and lung tissue homogenate (when enough volume was available after NTHi and IAV counts were conducted; *n* ≥ 3) (Fig. 4). All cytokines except for IL-10 were detectable in at least one of the sample types. Low or baseline titers were measured for IL-1β and IFN-γ in all tissue types (Fig. S3). Titers for IL-6 and KC in all tissue types are shown in Fig. 4b through g. IAV only and IAV + NTHi challenged mice had increased IL-6 in their nasal washes on Days 5 and 7 in comparison to the saline-treated group [Day 5 IAV vs saline: 7.55 (IQR: 5.25–54.48) pg/mL vs 0.34 (IQR: 0.34–0.34) pg/mL, respectively, *P* < 0.05; Day 5 IAV + NTHi vs saline: 125.9 (54.11–306.1) pg/mL vs 0.34 (0.34–0.34) pg/mL, respectively, *P* < 0.001] (Fig. 4b). By Day 7, the IL-6 response was still higher in nasal washes of the IAV and IAV + NTHi groups in comparison with the saline only group [Day 7 IAV: 125.9 (45.51–226.8) pg/mL, Day 7 IAV + NTHi: 72.72 (38.79–151.6) pg/mL vs Day 7 saline: 1.13 (0.34–2.41) pg/mL, *P* = 0.06 and *P* < 0.01, respectively]. The nasal wash KC responses were similar to those observed for IL-6 (Fig. 4c), with increased KC titers in the IAV and IAV + NTHi groups on Days 5 and 7 in comparison with the saline group [Day 5 IAV only = 25.61 (16.10–66.81) pg/mL, IAV + NTHi = 62.57 (25.76–135.6) pg/mL vs saline 1.04 (1.04–4.51) pg/mL, *P* < 0.05 and *P* < 0.001, respectively, Day 7 IAV only = 54.52 (39.31–117.5) pg/mL, IAV + NTHi = 140.5 (60.69–162.2) pg/mL vs saline 13.40 (2.32–21.66) pg/mL; *P* = 0.06 and *P* < 0.01, respectively]. The IAV + NTHi group had increased KC in the middle ear tissue by Day 7 compared to the saline group [81.15 (50.13–86.73) pg/mL vs 33.99 (33.54–36.90) pg/mL, respectively, *P* < 0.05].

Similar patterns were observed for KC in the lung tissue as in the nasal washes, with increased KC response in the lungs on Days 5 and 7 in the IAV only group compared with saline [Day 5 IAV only 68.67 (15.54–125.1] pg/mL vs saline 18.81 (14.05–23.07) pg/mL, *P* = 0.26 and Day 7 IAV 38.10 (17.39–72.91) pg/mL vs saline 10.16 (9.10–12.11) pg/mL, *P* < 0.05].

Of note is the absence of any cytokine signal in the specimens from the NTHi only group in comparison with the IAV only and IAV + NTHi groups (Fig. 4). Indeed, the IL-6 and KC responses in the NTHi only treatment group were often lower than the saline-treated group (Fig. 4). This was also observed for IFN-γ with decreased levels in the nasal wash of the NTHi only challenged mice compared to the saline only controls on Day 7 [9.59 (1.41–18.54) pg/mL vs 0.44 (0.44–0.44) pg/mL, *P* < 0.05 (Fig. S3b]. In the middle ear tissue and lungs, NTHi only challenged mice had a small but significant decrease in KC levels compared to saline-treated controls on Day 5 for lungs [18.81 (14.05–23.07) pg/mL compared to 12.46 (10.75–14.14) pg/mL] and Day 7 for middle ear tissue [33.99 (33.54–36.90) pg/mL compared to 31.63 (27.07–32.08) pg/mL] (*P* < 0.05 in all cases) (Fig. 4).

### Histological assessment of middle ear tissue reveals evidence of inflammation and infection

Haematoxylin and eosin (H&E) staining of fixed middle ear tissue was used for histological assessment of the middle ear tissue (Fig. S4). Potential thickening of the middle ear epithelium was observed behind the tympanic membrane of IAV + NTHi-treated mice [mean ± SEM epithelial thickness for saline: 190.5 ± 24.55 µm (*n* = 2), IAV: 208.8 ± 20.08 µm (*n* = 3), NTHi: 142.6 ± 56.71 µm (*n* = 2), and IAV + NTHi 265.1 ± 23.26 µm (*n* = 3)]. (No statistical analysis was conducted due to small sample size). Infiltrate was observed in the middle ear space of IAV + NTHi-treated mice (Fig. S4f), which was not observed in H&E stained sections of middle ear tissue from the other treatment groups.

## DISCUSSION

We have developed an infant mouse model of NTHi colonization and OM that reflects respiratory virus-driven bacterial OM in children (27, 30). Influenza challenge was essential for driving NTHi from the nose into the middle ear, as intranasal inoculation of NTHi only did not result in prolonged NTHi colonization or development of NTHi OM. Inflammation was observed in response to the development of NTHi OM in the nasal washes, and less so in the middle ear. Evidence of infiltration was observed in the ears of mice with NTHi OM. Disease development was the same between male and female mice, with no differences in clinical symptoms or bacterial/viral titers, permitting the use of both sexes in this model. The use of both sexes is important for providing safety and efficacy data for treatments that will be used in all children (in addition to meeting the principle of the 3Rs of animal research: reduce, refine, and replace).

NTHi does not naturally colonize any animal other than humans (9). This is one of the difficulties of developing suitable NTHi OM animal models. Chinchillas are one of the most widely used OM animal models due to the relatively large size of their tympanic membranes and bulla, allowing for easy dissection and direct injection of the pathogen of interest into the middle ear space (27). While this makes model development easier, there are several downsides including that transbullar injection does not mimic natural disease progression in humans (30), and that chinchillas are available only in North and South America, limiting global use of the model (27). The previously developed adult ascension mouse model of IAV driven NTHi infection of the middle ear does mimic human disease progression and uses a widely available lab animal—BALB/c mice. However, only half of the adult mice develop NTHi OM in this model (14), which makes testing of therapeutics for disease prevention and treatment difficult and requires larger group sizes to take this into account. Using the infant NTHi OM model, all mice developed NTHi OM by Day 5, which was sustained by Day 7 with clearance around Day 9. Levels of IL-6 in the nasal wash and middle ear tissue were approximately three-fold lower in the infant mice compared with the adult model on Days 5 and 7 (14); however, KC levels were similar. This suggests that while the infant mice were mounting a protective innate immune response, it was not as strong as observed in the adult mice and may contribute to the increased susceptibility to NTHi-driven OM in infant mice (and young children). With a 100% disease rate and longer duration of infection, this makes the infant mouse model much more suited for testing vaccines and therapeutics. Additionally, the dense and prolonged NTHi colonization of the infant mice was stable at least to Day 9 (we did not measure any later), providing a promising feature for testing the therapeutic impact on NTHi colonization—the precursor for all NTHi disease development.

In terms of the immune responses in this model, we measured the innate response for each sample collection day. IAV challenge was the main driver of the measured innate inflammatory response, which is likely to facilitate NTHi proliferation and dissemination into the ear - as is proposed to occur in children (14, 15). In the nasal wash, both IL-6 and KC were significantly elevated but only in groups that had been challenged with IAV. In the middle ear tissue, both IAV and NTHi were required for an increased KC response. For the lungs, IAV was also the main driver of the increased KC response. Both IL-6 and KC are innate chemoattractants in mice, responsible for immune cell recruitment (31, 32), suggesting a strong innate response to intranasal IAV challenge in both the nasal wash and lungs, and that co-challenge with NTHi is required to extend that response to the middle ear tissue. The observation of a suppressed local innate immune response to NTHi aligns with previous reports of NTHi displaying immune evasion techniques (9), including suppression of cytokine signaling (14, 33).

While IAV facilitated NTHi colonization and dissemination into the ear, NTHi infection did not increase the IAV titers measured in the lungs. Instead, decreased IAV titers were observed in the nasal washes of co-challenged mice in comparison with the IAV only challenged mice, indicating that NTHi challenge may interfere or suppress IAV infection. The mechanisms behind this require further investigation.

The observation of middle ear epithelium thickening and infiltrate in the middle ear space are in line with previous reports in OM mouse models of NTHi-OM (transbullar) and pneumococcal-OM (intranasal ascension driven by influenza) (34, 35). Transbullar inoculation of NTHi resulted in thickened middle ear epithelium and increased immune cell infiltrate in the middle ear space in adult *C57BL/6* mice (34). An IAV ascension model of pneumococcal-driven OM found increased infiltrate in the middle ear space of the infant mice (35). Similar results were observed in this infant mouse model for NTHi-driven OM, although small sample sizes mean no statistical testing could be performed.

Limitations of this infant NTHi OM model are (i) that only acute OM has been studied, not chronic or repeat OM [which is lacking across the whole OM research field (28)]; (ii) the model is labor intensive and requires an in-house breeding program and timed mating, and (iii) care must be taken to not overhandle the young mice to prevent rejection from the mothers.

In summary, we have developed an infant mouse model of viral-driven NTHi colonization and acute NTHi OM that can be used for development and testing of therapeutics targeted specifically at NTHi.

## MATERIALS AND METHODS

### Species, strains, and inoculum

#### Animals

All experiments were approved by the Telethon Kids Animal Ethics Committee prior to beginning the work (application: AEC #374). Specific pathogen-free mice (36 seven-week-old female BALB/cARC mice and 20 seven-week-old male BALB/cARC) were purchased from the Animal Resources Centre (Murdoch, WA, Australia) and housed at the Telethon Kids Institute under Physical Containment level 2 conditions and a 12:12 standard light/dark cycle. Standard mouse chow (Specialty Feeds, Glen Forrest, WA, Australia) and acidified water were freely accessible. After an acclimatization period of 1 week, male and female mice were housed together overnight for up to 2 weeks. Plugged female mice were separated and housed together until gestational day 14.5 (GD14.5) whereupon they were weighed. Mice weighing over 22 g were considered pregnant and housed in separate cages with extra bedding and red plastic huts provided for nesting. Starting at GD18.5, the presence of pups was checked daily to ascertain the day of birth. The average litter size was 3 for successful pregnancies, with a total of 79 pups from 24 litters from 8 rounds of breeding used in this study. An additional seven pups were used in NTHi dose optimization (see Fig. S2). To enable handling of manageable group sizes and control for day-to-day variability, litters of mice were distributed across treatment groups for each experiment (see Table S1 for litter allocation across experiments). When mice were 6 days old, their footpads were tattooed with green animal tattoo ink and tattoo device (Ketchum, Fine Science Tools, USA) using a 29-gauge needle to allow identification of individual mice. Mice were sexed by eye by trained personnel. Individual litters were assigned to one of four groups: saline-treated control, IAV challenge, NTHi challenge, or IAV + NTHi challenge. Multiple litters were used per treatment group.

#### Influenza

Influenza A/Mem71 virus (IAV) is a combined [A/Memphis/1/71 (H3N2) × A/Bellamy/42 (H1N1)] mouse-adapted H3N1 strain of influenza A (36) that causes mild to moderate disease symptoms in adult and infant mice (36, 37). IAV was subpassaged through Madin-Darby canine kidney (MDCK) cells (NBL-2, ATCC CCL-34) in Dulbecco’s modified Eagle’s medium (DMEM; Gibco, Sydney, Australia), then harvested from tissue culture supernatant and stored at −80°C. Viral titers after thawing from −80°C storage were determined by plaque assay as previously described (38).

#### NTHi

Genetically modified spectinomycin-resistant nontypeable *Haemophilus influenzae* (NTHi 2866 Spec^r^) was harvested mid-log growth phase as previously described (39), with the addition that 0.1 mg/mL spectinomycin was added to the culture media during growth to maintain resistance. This mutant strain was generated by allelic exchange of pseudogene R2866_1356 with a spectinomycin resistance cassette that was amplified from plasmid pR412 as previously described (14, 40).

### Infectious challenges

**Fig 5:**
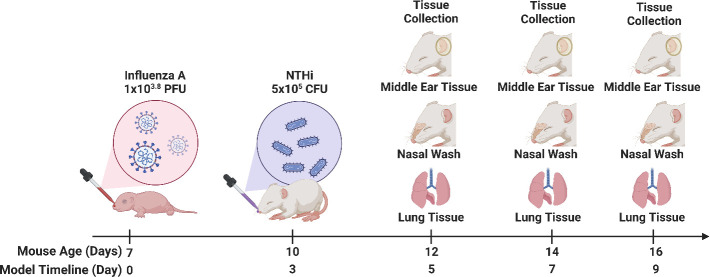
Experimental timeline for infant model of NTHi OM. Experimental Day 0 (IAV challenge) occurs when the mouse is 7 days old. Mice were given an inoculation of influenza A (IAV) (1 × 10^3.8^ PFU) or saline on Day 0. Three days following, mice were given an inoculation of NTHi (5 × 10^5^ CFU) or saline. Samples were collected on Days 5, 7, and 9 post-IAV inoculation. This image was created using Biorender.com.

At 7 days of age (Day 0 = IAV challenge), mice were intranasally challenged with either 5 µL of saline (phosphate-buffered saline, PBS) or 1 × 10^3.8^ PFU IAV in 5 µL of saline, calculated using previously described dose changes between adult and weaning age mice (41). Three days after the first challenge, mice were challenged a second time with either 5 µL saline or 5 × 10^5^ CFU NTHi in 5 µL of saline. Nasal washes and tissue samples were collected at Days 5, 7, and 9 days post-IAV challenge as outlined in Fig. 5. All challenges were performed without anesthetic due to the young age of the mice. Mice were monitored and weighed daily.

### Specimen collection and processing

Mice were euthanized after 5 min in a small chamber containing 5% isoflurane (SomnoSuite, Ken Scientific). Nasal washes [50 µL saline lavaged into the nasal space through a 30-gauge Rycroft anterior chamber cannula (Sterimedix ophthalmic, IQmedical)], middle ear tissue (dissected from the skull) and whole lungs were collected (Fig. 5) immediately post-mortem and stored on ice. Samples were homogenized in saline (250 µL for middle ear tissue and 1 mL for lung tissue), serially diluted 10-fold, and spotted onto chocolate agar plates (PathWest, Perth, Australia) overlayed with spectinomycin (200 µL of 10  mg/mL spectinomycin) to select for NTHi Spec^r^ and incubated (37°C, 5% CO_2_) overnight. Viable counts were conducted by personnel not involved in the animal model to ensure blinding. The limit of detection (LOD) was 1.92 Log_10_ CFU/mL. For ease of statistical analysis, all values below the limit of detection were replaced with a value half that of the LOD.

### Viral copy assessment

Influenza virus qPCR was performed as described previously (35). In brief, RNA was extracted using TRIzol (Invitrogen, USA), purified using an RNAeasy minielute kit (Qiagen, Germany), and treated with DNase (Turbo DNAse; Ambion, USA). Synthesis of cDNA was performed using random oligo(dT)_18_ (Roche) and Transcriptor High Fidelity cDNA synthesis kit (Roche, Switzerland). Quantitative PCR was performed using SYBR green (Quantance) and forward and reverse primers for the Influenza A/Mem71 Matrix gene (42) (Forward primer sequence 5′-AAGACCAATCTTGTCACCTCTGA-3′, Reverse Primer Sequence 5′-TCCTCGCTCACTGGGCA-3′). Quantitative viral copy number was determined using 10-fold dilutions of a plasmid containing the Influenza A/Mem71 matrix gene.

### Immune mediator analysis

Aliquots of nasal wash, and homogenized middle ear and lung samples were centrifuged at 13,000 RPM for 10 min at 4°C. Supernatant was collected and filtered through 0.2 µm filters (MERK) before storage at −80°C. Once thawed, immune mediator response for IL-1β, IL-6, IL-10, IFN-γ, and KC was assessed using custom-generated multiplex kits from Bio-Rad (X-Plex kit, Bio-Rad, USA), following kit protocol. Analysis of multiplex results was performed using a Bioplex 200 with Bioplex Manager software V6.1.1. For ease of statistical analysis, all values below the limit of detection were replaced with a value half that of the lowest standard (limit of detection for IL-1β, IL-6, IFN-γ, and KC were 1.39, 0.67, 0.87, and 2.07 pg/mL, respectively).

### Histology

Whole infant mouse heads obtained on Day 7 post-IAV challenge were fixed for at least 24 h in 10% formalin, prior to embedding in paraffin (*n* = 3 per group). Mouse heads were processed overnight for paraffin infiltration and embedded for horizontal sections beginning from the base of the skull. The head was cut until the outer ear canal was visible and then 10 µm sections were taken at intervals of 125 µm to isolate the middle and inner ear. Sections were then stained with H&E and slides where the tympanic membrane could be positively identified were selected for imaging using a Panoramic MIDI scanner and paired software (3DHISTECH Ltd.).

### Statistical analysis

Statistical analysis was performed using GraphPad Prism software (V9.0.0). Analysis between groups was conducted using two-way Mann-Whitney testing methodologies due to the non-parametric nature of the data. All data are described as median or median (IQR) except for weight which is described as mean ± SD and epithelial thickness measurements described as mean ± SEM.

## Data Availability

The data that support the findings of this study are available from the corresponding author upon request.

## SUPPLEMENTAL MATERIAL

Supplemental material iai.00453-23-s0001.docx Fig. S1 to S4; Table S1.

ASM does not own the copyrights to Supplemental Material that may be linked to, or accessed through, an article. The authors have granted ASM a non-exclusive, world-wide license to publish the Supplemental Material files. Please contact the corresponding author directly for reuse.
