# Supplementary material for: An infant mouse model of influenza-driven nontypeable Haemophilus influenzae colonization and acute otitis media suitable for preclinical testing of novel therapies
Source: Infect Immun. 2024 Apr 11;92(5):e00453-23. doi: 10.1128/iai.00453-23 (PMC11075455; doi:10.1128/iai.00453-23)
Supplement: Supplemental material — Fig. S1 to S4; Table S1. [file iai.00453-23-s0001.docx]

**Supplementary Figure 1**

**Figure S1: No sex specific differences in NTHi or viral titres in nasal wash, middle ear or lungs in NTHi OM model.**

Nontypeable *Haemophilus influenzae* (NTHi) density in CFU/mL from a) nasal washes, b) middle ear tissue homogenates and c) lung homogenates and influenza A viral titre from d) nasal washes, e) middle ear tissue homogenates and f) lung homogenates of mice on Days 5, 7 and 9 post influenza A virus (IAV). Each dot represents an individual mouse, females in blue and males in green. Horizontal bars depict the median NTHi density, and the dashed line represents the limit of quantification. *, P < 0.05; **, P < 0.01; ***, P < 0.001.

**Supplementary Figure 2**

**Figure S2: Weight and NTHi titres between two different NTHi challenge doses.**

a) Percent weight change (mean ± SD) of infant mice challenged with IAV + NTHi at a dose of either 5x10^5^ CFU or 5x10^6^ CFU NTHi (with saline treated mice as a control). b-c) Nontypeable *Haemophilus influenzae* (NTHi) density in log10 CFU/mL from b) nasal washes and c) middle ear tissue homogenates on Day 5 and Day 7 post IAV for 5x10^5^ CFU NTHi (purple) and 5x10^6^ CFU NTHi (green). Each dot represents an individual mouse. Horizontal bars depict the median NTHi density, and the dashed line represents the limit of quantification.

**Supplementary Figure 3**

**Figure S3: IL-1β and IFN-γ response in nasal wash, MET and lungs** **on Days 5 and 7 post IAV challenge.**

Inflammatory mediator levels in a-b) nasal washes, c-d) middle ear tissue homogenate and e-f) lung homogenates from mice on Days 5 and 7 post influenza A (IAV) or saline control challenge. Each dot represents an individual mouses cytokine release in pg/mL, horizontal bars depict median analyte titre and dashed line represents the limit of quantification. *, P < 0.05; **, P < 0.01; ***, P < 0.001.

**Supplementary Figure 4**

**
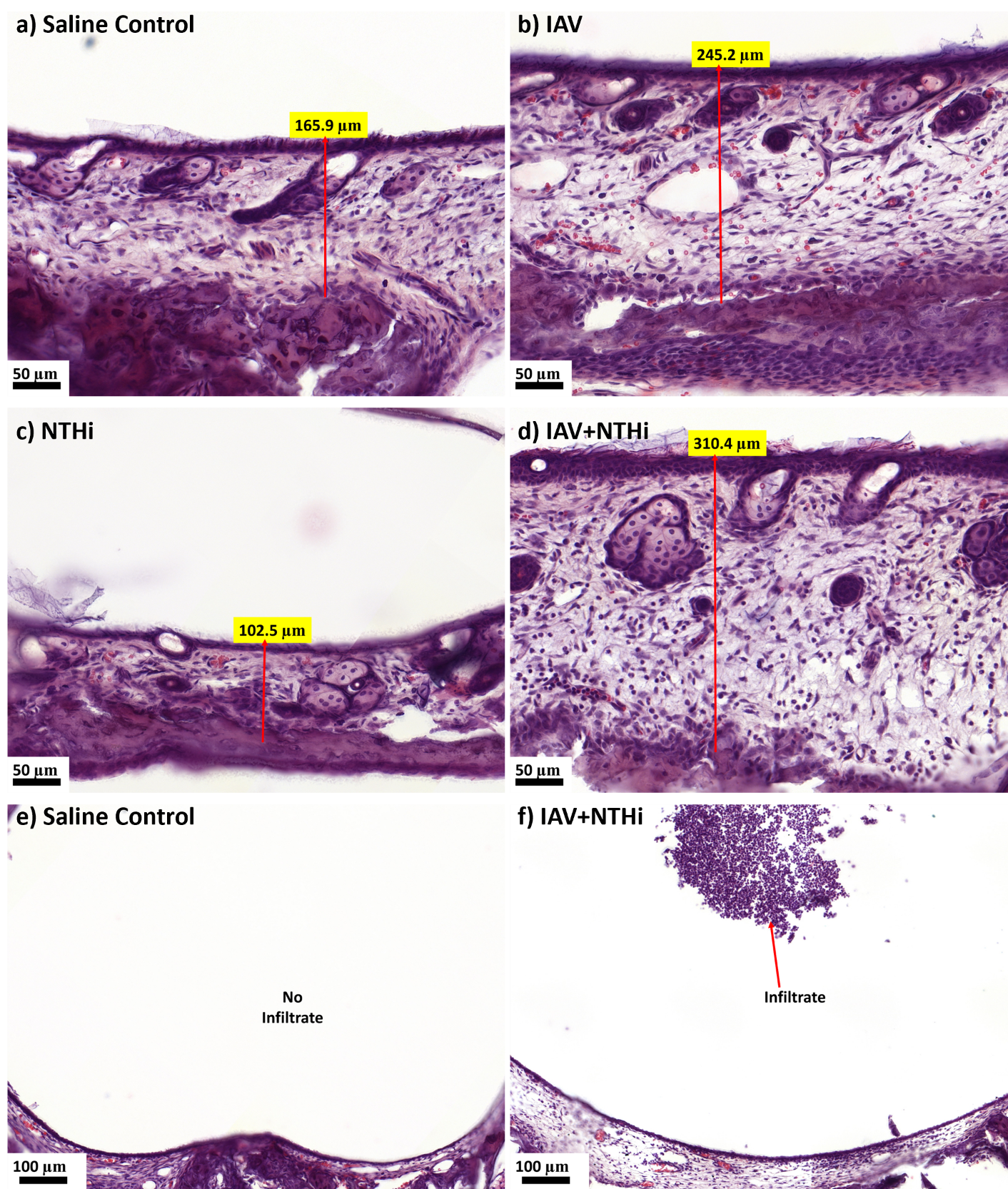
Figure S4: Histological images of middle ear tissue sections on Day 7 post IAV challenge.**

Images of the middle ear epithelium are shown for a) saline treated control mice, b) IAV challenged mice, c) NTHi challenged mice and d) IAV+NTHi challenged mice. Images of the middle ear space were assessed for infiltrate. Infiltrate was only found in the IAV+NTHi treated mice. Comparison images of the middle ear space are shown for e) saline treated control mice and f) IAV+NTHi challenged mice. Numbers in yellow boxes are the measured epithelial thickness for each mage. Images a-d were taken a 25x magnification, images e-f at 12x magnification. Scale bar at 50 or 100 µm.

**Supplementary Table 1**

**Table S1: Demonstration of allocation of litters to treatment groups.** Litters in the same breeding round were bred and used concurrently and thus grouped together, showing that data for each treatment group was obtained over multiple experiments and litters.

|  |  | **Day 5** | | | | **Day 7** | | | | **Day 9** | | | |
| --- | --- | --- | --- | --- | --- | --- | --- | --- | --- | --- | --- | --- | --- |
| Round | Litter # | Saline | IAV | NTHi | NTHi + IAV | Saline | IAV | NTHi | NTHi + IAV | Saline | IAV | NTHi | NTHi + IAV |
| Breeding round 1  (February 2022) | 1 |  | 1 |  |  |  | 1 |  |  |  | 1 |  |  |
|  | 2 |  | 2 |  |  |  | 1 |  |  |  | 1 |  |  |
|  | 3 |  |  | 1 |  |  |  | 1 |  |  |  |  |  |
|  | 4 |  |  | 2 |  |  |  |  |  |  |  | 1 |  |
|  | 5 | 3 |  |  |  |  |  |  |  |  |  |  |  |
|  | 6 |  |  |  |  |  |  | 1 |  |  |  |  |  |
|  | 7 |  |  |  |  |  | 2 |  |  |  | 2 |  |  |
| Breeding round 2 (April 2022) | 8 |  |  |  |  | 1 |  |  |  |  |  |  |  |
|  | 9 |  |  | 1 |  |  |  | 1 |  |  |  | 2 |  |
|  | 10 |  |  |  | 3 |  |  |  | 2 |  |  |  |  |
|  | 11 |  |  |  | 2 |  |  |  | 2 |  |  |  |  |
| Breeding round 3 (May 2022) | 12 |  |  | 2 |  |  |  | 2 |  |  |  |  |  |
|  | 13 | 3 |  |  |  | 3 |  |  |  |  |  |  |  |
|  | 14 |  |  |  | 2 |  |  |  | 2 |  |  |  |  |
|  | 15 |  |  | 1 |  |  |  | 1 |  |  |  |  |  |
| Breeding round 4  (July 2022) | 16 |  |  |  | 3 |  |  |  | 2 |  |  |  |  |
| Breeding round 5  (Sept 2022) | 17 |  |  |  |  |  |  |  |  |  |  |  | 5 |
| Breeding round 6  (Oct 2022) | 18 |  |  |  |  |  |  |  |  | 3 |  |  |  |
| Breeding round 7  (November 2022) | 19 |  |  |  |  | 2 |  |  |  |  |  |  |  |
|  | 20 |  |  |  |  |  | 3 |  |  |  |  |  |  |
|  | 21 |  |  |  |  |  |  | 2 |  |  |  |  |  |
|  | 22 |  |  |  |  |  |  |  | 3 |  |  |  |  |
| Breeding round 8  (Mar 2023) | 23 |  | 1 |  |  |  | 2 |  |  |  |  |  |  |
|  | 24 |  | 2 |  |  |  | 1 |  |  |  |  |  |  |
| TOTAL/group |  | 6^a^ | 6^ab^ | 7 | 10 | 6^ac^ | 10^abd^ | 8^c^ | 11^d^ | 3 | 4^a^ | 3 | 5 |

^a^ 3 mice/group had cytokine responses measured in nasal washes and middle ear tissue (Figure 4).

^b^ 3 mice/group had influenza viral copy numbers measured in nasal washes and middle ear tissue (Figure 3).

^c^ 2 mice/group used for histology analysis (Figure S4).

^d^ 3 mice/group used for histology analysis (Figure S4).
